# Parainfluenza 3 Respiratory Infection Associated with Pericardial Effusion in a Very Low Birthweight Infant

**DOI:** 10.1155/2017/5687490

**Published:** 2017-10-24

**Authors:** Cristina Aranda Cazón, Luis Arruza Gómez, Gloria Herranz Carrillo, Cristina González Menchén, Zarife Daoud Pérez, José Antonio Martínez-Orgado, José Tomás Ramos Amador

**Affiliations:** ^1^Department of Pediatrics, Hospital Clínico San Carlos, Instituto de Investigación Sanitaria del Hospital Clínico San Carlos (IdISSC), Madrid, Spain; ^2^Division of Neonatology, Hospital Clínico San Carlos, Instituto de Investigación Sanitaria del Hospital Clínico San Carlos (IdISSC), Madrid, Spain

## Abstract

Parainfluenza 3 virus is a frequent cause of respiratory infections in the pediatric population although it is uncommonly diagnosed in neonates, being usually reported as neonatal intensive care unit microepidemics. We report a case of parainfluenza 3 respiratory infection associated with pericardial effusion in a very low birthweight infant.

## 1. Case Report

We present the case of a male neonate born at week 25 of gestational age and weighed 720 grams. During pregnancy follow-up, the mother received labetalol because of gestational hypertension. Ultrasound controls during pregnancy were normal, and routine serologies were negative.

At week 25 of gestation, severe preeclampsia was diagnosed. Fetal ultrasound showed intrauterine growth retardation type 1 and redistribution in Doppler flow. Caesarean delivery was indicated due to severe preeclampsia after sulfate magnesium administration for neuronal protection and complete pulmonary maturation. The newborn needed intubation in the delivery room with 100% fraction of inspired oxygen (FiO_2_) and intratracheal instillation of surfactant. Then, he was extubated and connected to nasal continuous positive airway pressure (CPAPn). When FiO_2_ was decreased to 30%, the patient was transferred to the neonatal intensive care unit (NICU).

**Figure 1:**
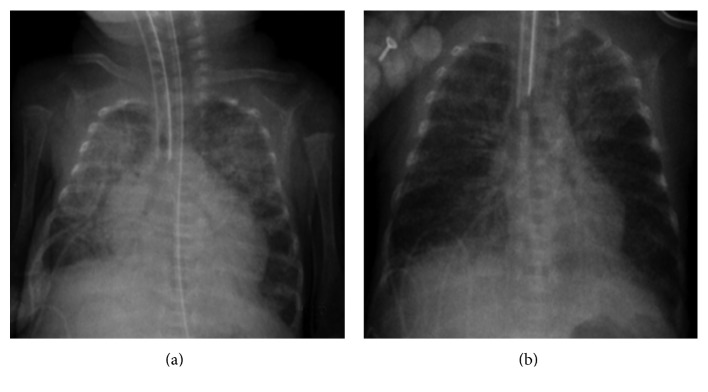
Acute (a) and convalescent (b) chest radiographs demonstrating cardiomegaly at the time of the clinical deterioration, with near complete resolution five days later.

**Figure 2:**
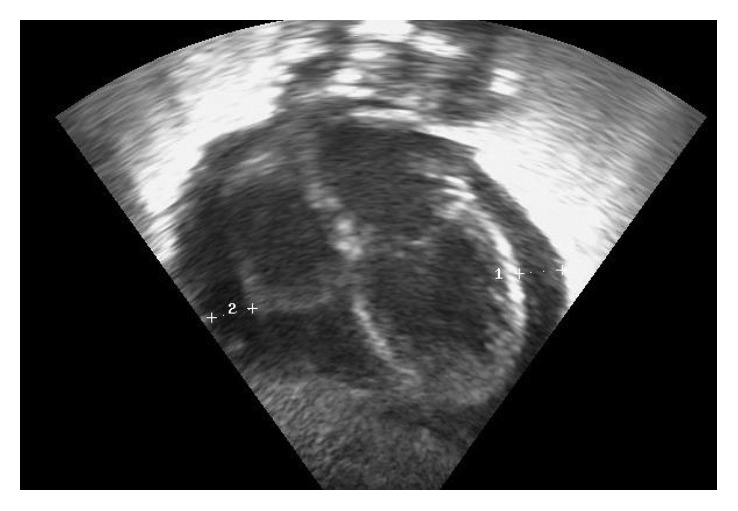
Echocardiography showing pericardial effusion. Dimensions: 1 = 0.43 cm; 2 = 0.41 cm.

At 29 week postconceptional age, the infant was stable with noninvasive ventilation (mean airway pressure (MAP) 6 cmH_2_O, FiO_2_ 0.40). Suddenly, clinical deterioration occurred, involving respiratory distress, increased needs of oxygen therapy, tachypnea, bradycardia, desaturations, and frequent apnea episodes, that required intubation and mechanical ventilation (MAP 12 cmH_2_O, FIO_2_ 0.50). Laboratory tests evidenced lymphocytosis and hypertransaminasemia. Blood culture, urine culture, and cerebrospinal fluid culture were negative (Table 1). Chest X-ray showed bilateral pulmonary infiltrates and significant cardiomegaly (Figure 1()). Echocardiography revealed diffuse pericardial effusion involving predominantly right chambers (Figure 2()). Contractility and right and left cardiac outputs were preserved as shown in Table 1.

Polymerase chain reaction (PCR) in nasopharyngeal swab was requested the day after the clinical deterioration and was positive for parainfluenza 3 virus (PIV3). The multiplex PCR used was CLART-PneumoVir. It detects 17 respiratory viruses: respiratory syncytial viruses (RSV); RSV group-A; RSV group-B; PIV1; PIV2; PIV3; PIV4; coronaviruses; human metapneumovirus; adenoviruses; enteroviruses; influenza A (including H3N2 and both seasonal and pandemic H1N1), B, and C viruses; rhinoviruses; and bocavirus. Because newborn family members and neonatal intensive care unit staff were asymptomatic before, during or after the episode, nasopharyngeal swabs could not be obtained from the contacts. Other infectious causes of pericardial effusion were incompatible with the clinical picture: the patient was not treated with drugs that may had caused pericardial effusion, there was no possible traumatic cause (central venous catheter perforation and penetrating trauma), and there were not clinical, analytical, or radiological signs of other causes (tuberculosis, fungal infections, collagen diseases, etc.). Blood culture for other microorganisms, including bacteria and fungi, was negative. Because of this, PIV3 may have been the trigger for the pericardial pathology.

Due to the hemodynamic stability of the patient, a conservative approach was adopted. The patient did not require anti-inflammatory or diuretic therapy to reduce pericardial effusion. Serial echocardiograms and chest X-ray confirmed its reduction until its complete resolution within 5 days (Figure 1()). Figure 1() shows the cardiomegaly at the clinical deterioration, with near complete resolution. At 36 weeks postconceptional age, the patient developed severe bronchopulmonary dysplasia (BPD), requiring high-flow nasal cannulae and 40% oxygen supplementation.

## 2. Discussion

To our knowledge, there are only two cases reported of pericardial effusion associated with parainfluenza viruses in children and none of them in the neonatal period. The first one describes an 8-month-old boy diagnosed with pneumonia and severe combined immunodeficiency. Parainfluenza 3 was isolated in respiratory secretions and pericardial and cerebrospinal fluids. Postmortem evaluation confirmed the presence of the pathogen in the lung, brain, and pericardial samples [1]. The second case is a 9-year-old immunocompetent patient with pericardial effusion in whom parainfluenza 4 was isolated in nasopharyngeal aspirate. The patient was successfully treated with ibuprofen [2]. There are two other cases reported in adults. In the first one, parainfluenza 3 viral infection was considered to be the cause of a fulminant myocarditis with pericardial effusion in a 35-year-old woman with diabetic ketoacidosis and type 1 diabetes [3]. The second one described a 62-year-old woman with pneumonia and pericardial effusion as a result of infection with parainfluenza virus type 4 and thrombotic thrombocytopenic purpura [4].

Parainfluenza virus is an RNA virus belonging to the Paramyxoviridae family, with four types described. It is a prevailing cause of viral respiratory infection in the pediatric population after respiratory syncytial virus, enteroviruses, or human metapneumovirus. Although respiratory viral infections are usual among hospitalized newborns, they are not clinically suspected because the signs and symptoms of infection are nonspecific or too subtle. For this reason, they are seldom diagnosed in the neonatal intensive care unit. There are scarce reports presenting isolated cases like this one as most of the published data refer to neonatal intensive care unit microepidemics in which parainfluenza 3 was just one of the different viruses detected [5]. Those outbreaks are thought to be related to longer stay in the neonatal intensive care unit. As the main route of transmission of this pathogen is through infected secretions, respiratory and contact isolation is essential to prevent disease outbreaks.

Premature infant population may develop parainfluenza virus infection with atypical or even clinically unapparent signs and symptoms [6] as in our patient. Clinical signs are nonspecific (mainly respiratory with increased secretions, respiratory distress, coughing, tachypnea, pneumonia, or apnea episodes). Diagnosis requires a high index of suspicion in the presence of suggestive symptoms, since other infecting viruses such as respiratory syncytial viruses, enteroviruses, rhinoviruses, or coronaviruses may induce similar clinical pictures confirmation. Diagnosis is based on the detection of virus-specific RNA using nucleic acid amplification techniques from a respiratory sample such as a nasopharyngeal swab. This technique has been a breakthrough in the diagnosis as it could decrease unnecessary use of empirical antibiotic therapy. Other diagnostic methods are viral culture, usually less available and with longer turnaround time results, and direct viral detection in respiratory secretions by immunofluorescence or enzyme immunoassay, which shows lower sensitivity and specificity than PCR. Currently, there is no specific treatment against parainfluenza virus infections. Management of the infected patient is based on supportive therapy according to the clinical condition.

The main limitation of our case report is the unconfirmed etiological presence of parainfluenza virus in pericardial effusion. Detection of parainfluenza 3–specific RNA from pericardial fluid or blood would offer direct evidence that PIV3 caused the effusion. Pericardial fluid was not obtained due to the invasiveness of the technique in the context of clinical and hemodynamic stability of our patient and spontaneous resolution in 5 days.

In conclusion, parainfluenza 3 infection should be considered during the diagnostic evaluation of neonatal pericarditis. This may prevent possible severe complications and reduce unnecessary use of antibiotics. High index of suspicion is required for rapid diagnosis and appropriate management.

## Figures and Tables

**Table 1:** Diagnostic tests performed.

Test	Results
Blood test before the diagnosis of the pericardial effusion, during sudden clinical deterioration	Leukocytosis 15.08 cells/mm^3^ (5–14.5 cells/mm^3^; 75% lymphocytes, 12% neutrophils); hemoglobin 12.5 g/dl (10–13 g/dl) and platelet count 280/mm^3^ (250–450/mm^3^). ALT 203 U/L (6–50 U/L), AST 180 U/L (35–140 U/L), C-reactive protein (CRP) 3, 4 mg/dl (<2 mg/dl); procalcitonine 0.3 ng/ml (<0.5 ng/ml)

Blood culture	Negative

Urine culture	Negative

Cerebrospinal fluid culture	Negative

Chest X-ray	Bilateral pulmonary infiltrates and significant cardiomegaly

Echocardiography	Diffuse pericardial effusion affecting predominantly right chambers. Ejection fraction: 76%; shortening fraction: 41%; LVO: 355 ml/kg/min; RVO: 395 ml/kg/min

PCR in nasopharyngeal swab	Positive for parainfluenza 3 virus

Blood tests after the resolution of the pericardial effusion	Leukocytes 9.8 cells/mm^3^(74.4% lymphocytes, 15% neutrophils); hemoglobin 13.1 g/dl and platelet count 255/mm^3^. ALT 89 U/L/AST 73 U/L, C-reactive protein (CRP) 1.7 mg/dl; procalcitonine 0.07 ng/ml

## Abbreviations

BPD: Bronchopulmonary dysplasia
FIO_2_: Fraction of inspired oxygen
LVO: Left ventricular output
NICU: Neonatal intensive care unit
PCR: Polymerase chain reaction
MAP: Mean airway pressure
RVO: Right ventricular output.
